# Risk adjustment for cesarean delivery rates: how many variables do we need? An observational study using administrative databases

**DOI:** 10.1186/1472-6963-13-13

**Published:** 2013-01-10

**Authors:** Elisa Stivanello, Paola Rucci, Elisa Carretta, Giulia Pieri, Maria P Fantini

**Affiliations:** 1 Department of Medicine and Public Health, University of Bologna, via San Giacomo 12, Bologna, 40126, Italy

## Abstract

**Background:**

Various studies indicate that inter-hospital comparisons have to take case mix into account and that risk adjustment procedures are necessary to control for potential predictors of cesarean delivery (CD). Different data sources have been used to retrieve information on potential predictors of CD. The aim of this study was to compare the discrimination capacity and fit of predictive models of CD created using different sources and to assess whether more complex models improve inter-hospital comparisons.

**Methods:**

We created 4 predictive models of CD. One model included only variables from Hospital Discharge Records of the index hospitalization, one included also information from previous hospitalizations, one also clinical variables from birth certificates (BC) and one also socio-demographic variables. We compared the four models using the Receiver Operator Curve and the Akaike and Bayesian Information Criteria.

**Results:**

Information from Birth Certificates improved the discrimination and model fit. Adding socio-demographic variables or past comorbidities did not improve the discrimination capacity or the model fit. Hospital-specific CD resulting from the models were highly correlated.

**Conclusions:**

Record linkage improves the performance of the models but does not affect inter-hospital comparisons.

## Background

Since the eighties, the World Health Organization (WHO) has recommended not to exceed a cesarean rate of 10–15% [1]. However, more recently, the WHO stated that, ‘both very low and very high rates of caesarean section can be dangerous, but the optimum rate is unknown’ and that ‘there is no empirical evidence for an optimum percentage or range of percentages, despite a growing body of research that shows a negative effect of high rates.’ In the absence of a optimum rate, it is worthwhile to compare cesarean delivery (CD) rates across hospitals in order to identify the birth units that mostly deviate from average values and decide where most efforts should be made to improve the quality and safety of birth [2].

Hospitals can be compared in terms of overall or primary CD rates. Primary CD rates are calculated after excluding women with a previous CD, who are at a very high risk for another cesarean section [3-5]. Moreover, various studies indicated that inter-hospital comparisons have to take case mix into account [6-11]. Risk adjustment procedures are necessary to control for potential confounders (socio-demographic and clinical risk factors of the mother and the fetus) that are not homogeneously distributed among different hospital populations.

Various data sources have been used to determine the overall or primary CD rate and retrieve information on potential predictors of CD. Some authors used only one data source, i.e. medical records [6] or hospital discharge records (HDR) [11-14] that, in some countries, such as the UK, include a maternity tail containing additional information on the pregnancy [11]. Other authors used multiple data sources linked to each other: maternal HDR linked to infant HDR [9,15], maternal HDR linked to clinical records [16] or to birth certificates (BC) [10] or maternal HDR linked to both infant HDR and BC [3,5,17].

Socio-demographic factors and clinical variables such as fetal weight and parity are usually retrieved from BC because here they are more complete, whereas co-morbidities of the mother are more frequently retrieved from HDR of the index hospitalization or of previous hospitalizations [3,9,10]. Database linkage entails the availability of more information but linkage procedures may be cumbersome, unfeasible or the proportion of records linked over the total records may be very low [18]. Empirical data from other clinical populations, such as general and cardiac surgery, reveal that most risk prediction comes from a relatively small number of variables [19,20]. To our knowledge no study explored this issue in relation to cesarean delivery. This study aimed to determine whether using one instead of two data sources and reducing the number of covariates impact CD risk prediction and affect inter-hospital comparisons of primary CD rates.

## Methods

The study was carried out in Emilia Romagna, a region located in North-Eastern Italy with about 4.4 million inhabitants and approximately 40,000 births per year. The national health service is statutorily required to guarantee the uniform provision of comprehensive care throughout the country. Essential health services are provided free of charge, or at a minimal charge. Local Health Authorities, that roughly coincide with administrative units (provinces) are responsible for the overall health of, and for the services offered to the population.

The HDR of the women who delivered in the Emilia Romagna from 1st January 2007 to 30th June 2009 were linked to the BC using the HDR identification code, the hospital code and the year of delivery.

The HDR include demographics (ID number, sex, date and place of birth, place of residence), discharge ID, admission and discharge dates, discharge diagnoses and procedures (International Classification of Diseases, 9^th^ Revision, Clinical Modification ICD-IX-CM), ward(s) of hospitalization, date(s) of in-hospital transfer, and the regional code of the admitting facility.

BC include demographic data of the mother, information on presentation and multiple pregnancy (singleton cephalic, singleton breech, transverse or oblique lie, etc.), parity (nulliparous, multiparous), the course of labour and delivery (spontaneous labour, induced labour or CS before labour) and gestational age (defined as the number of completed weeks at the time of birth) and other information on the newborn.

HDR were identified by using DRG codes (370–375), diagnosis (ICD-9-CM 640.xy - 676.xy (y = 1,2), V27 ) and procedure codes (ICD-9-CM 72.xy - 74.xy).

Records of women with a previous cesarean (ICD-9 CM code 654.2), discharged from hospitals without an operating room or small hospitals (<150 deliveries per year) or with a diagnosis of intrauterine death or still births (ICD-9 CM codes 656.4 V27.1, V27.4, V27.7) were excluded.

Primary CD were defined using procedure codes (ICD 9CM codes: 74.0, 74.1, 74.2, 74.4, 74.99 ) or diagnosis codes (ICD-9-CM 669.7) or DRG codes (370–371) or one BC variable (type of delivery).

We created 4 predictive models for primary CD (the Additional file 1 shows all variables of the four models and sources of information) using logistic regression:

· Model 1 included age and maternal comorbidities and information about delivery recorded in the HDR of the index hospitalization

· Model 2 included, in addition, past comorbidities recorded in HDR of hospitalizations occurred two years before delivery

· Model 3 included additional clinical variables retrieved from the BC

· Model 4 included also socio-demographic variables retrieved from the BC.

We applied a backward stepwise procedure to model 4 to identify the subset of variables significantly associated with caesarean section. Adjusted Odds ratios (OR) with 95% Confidence intervals (CI) were calculated for all models.

We evaluated the models at both patient and hospital level by comparing the full model (model 4) including variables derived from the two data sources with more parsimonious models, following the procedure of Dimick et al. [20]. In particular, to evaluate patient level risk prediction we used:

· the Receiver Operating Characteristic (ROC) to assess how well the model discriminates between women with and without a CD. The area under the curve ranges from 0.50 (no ability to discriminate) to 1 (perfect discrimination).

· the AIC (Akaike Information Criterion) and BIC (Bayesian Information Criterion), to obtain measures that combine fit and complexity of the model. Lower values indicate a better fit of the model taking complexity into account.

To evaluate hospital level risk adjustment, we compared CD measures obtained using the 4 models. To this purpose, we calculated the ratio of observed to expected CD (“O/E ratio”) at each hospital. The O/E ratio was calculated using logistic regression to predict a probability of CD (i.e., the expected outcome) for each woman. These probabilities were then summed for every hospital. The observed number of events was divided by the expected number, to obtain a risk-adjusted estimate of the outcome of interest; an O/E ratio of 1.0 is as expected given that hospital’s women characteristics, less than 1.0 is better than expected and greater than 1.0 is worse than expected. We calculated O/E ratios using the 4 models to estimate the expected CD.

We then analysed the correlations between the hospital O/E ratios obtained with different models. A correlation coefficient of 1.0 implies perfect agreement in O/E ratios [20].

## Results

We identified 102695 HDR and 100946 BC. 97% of the HDR were linked with BC (99626). After applying inclusion and exclusion criteria we obtained 87849 deliveries and a study population of 87574 women with complete data for the variables of interest in 29 hospitals. The primary cesarean section rate was 22.36%. The number of variables that were left in the models was: 21 in model 1 and 2, 26 in model 3 and 29 in model 4. Additional file 2 shows the characteristics of the study sample and the adjusted OR of CD for all selected variables in the four models. Across all models, a maternal age lower than 30 years was associated with a lower likelihood of CD, with aOR ranging from 0.34 to 0.88, while an age higher than 34 years was associated with an increased likelihood of CD, with aOR ranging from 1.18 to 2.36. Other demographic characteristics, included in model 4, contributed to predicting CD. In particular, a citizenship in high-income countries, compared to Italian citizenship, was associated with a 27% reduced likelihood of CD (aOR=0.73, 95% CI 0.59-0.91). Middle school education and University-level education were associated with a 14% and 18% reduced likelihood of CD compared to high-school education and a non-declared marital status was also associated with a 11% reduction in the likelihood of CD compared with being married.

Cord prolapse, malpresentation, placenta praevia, placental abruption or uterine hemorrhage, HIV and multiple births, were the risk factors associated with the highest risk of CD in all models.

**Figure 1:**
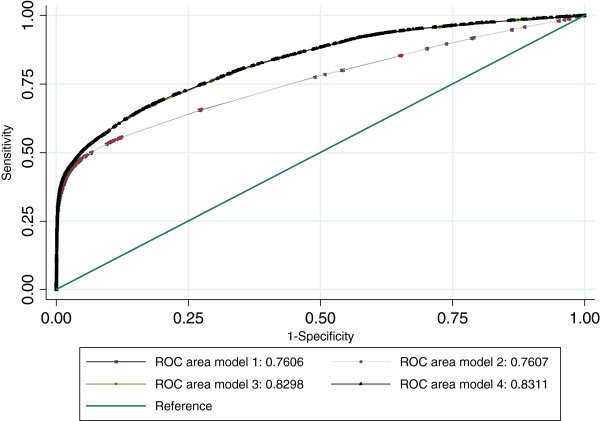
Receiver operating characteristic curves of the four models.

Table 1 shows the patient level discrimination capacity and the model fits. The areas under the ROC curves ranged from 75.89% in model 1 to 83.11% in model 4, with significant differences between models 2 and 3 and almost no differences between models 1 and 2 and between models 3 and 4 (Figure 1). In terms of goodness of fit and taking complexity into account, model 3 and 4 performed better than model 1 and 2, with only small differences between models 1 and 2 and between models 3 and 4. When considering the hospital level risk prediction, the hospital O/E ratios obtained with different models (Table 2) were highly correlated with each other. The correlation between model 1 and 2 was 1, between model 3 and 4 was 0.99 and between model 2 and 3 was 0.95.

**Table 1:** Discrimination and calibration capacity and goodness of fit of the four predictive models

	**Model 1**	**Model 2**	**Model 3**	**Model 4**
**ROC**	75.89	75.90	82.98	83.11
(95% CI)	(75.62–76.49)	(75.63–76.50)	(82.63–83.32)	(82.76–83.45)
**AIC**	69321.11	69325.12	64048.5	63975.75
**BIC**	69565	69569.01	64367.43	64379.1

**Table 2:** Hospital volumes and O/E ratios

		**O/E ratios**			
**hospital**	**n. deliveries**	**Model 1**	**Model 2**	**Model 3**	**Model 4**
1	3000	1.23	1.22	1.25	1.25
2	1710	1.22	1.22	1.25	1.24
3	1513	0.98	0.98	1.01	1.00
4	473	1.30	1.30	1.40	1.40
5	1869	0.98	0.98	1.09	1.07
6	1914	0.97	0.97	1.02	1.02
7	1558	1.18	1.18	1.25	1.24
8	510	1.39	1.38	1.44	1.43
9	3735	0.68	0.68	0.71	0.71
10	1364	0.83	0.83	0.84	0.82
11	1085	0.66	0.66	0.74	0.73
12	2501	1.09	1.08	1.09	1.08
13	6408	0.92	0.92	0.89	0.89
14	2315	1.16	1.16	1.15	1.15
15	1289	0.98	0.99	0.99	1.05
16	1687	0.69	0.69	0.74	0.79
17	3271	1.05	1.05	1.02	1.02
18	2337	0.76	0.76	0.78	0.78
19	2033	0.77	0.77	0.79	0.79
20	3456	1.04	1.04	1.05	1.04
21	4837	0.75	0.75	0.76	0.76
22	6195	1.04	1.04	0.98	0.98
23	2370	0.98	0.98	1.05	1.05
24	781	1.41	1.40	1.35	1.34
25	5520	1.17	1.17	1.17	1.17
26	5402	0.89	0.89	0.89	0.88
27	7240	1.03	1.03	1.06	1.06
28	7916	1.18	1.18	1.12	1.13
29	3285	0.91	0.92	0.87	0.87

We carried out secondary analyses excluding from the models preterm deliveries, women with herpes or antepartum hemorrhage/ placental abruption/placenta previa or malpresentations. The areas under the ROC curves for the 4 models ranged from 64.23% to 75.02% denoting a poorer discrimination capacity of the remaining variables to discriminate cesarean section. Results confirmed that models 3 and 4 were almost equivalent and performed better than models 1 and 2. The correlations of the hospital O/E ratios obtained with the four models ranged from 0.99 to 1.

## Discussion

Our study shows that all the four models have a high discrimination capacity but models including variables retrieved from both BC and HDR show a higher discrimination capacity than the ones including only variables from the HDR. In addition, the models including variables from both BC and HDR show a better fit taking complexity into account. Socio-demographic variables, such as maternal education, citizenship and maternal status, or variables derived from previous hospitalizations did not modify the discrimination capacity or the fit of the models substantially.

The discrimination capacity of our predictive models is in line with previously reported findings [6,7,11,16] even if other studies including only clinical risk factors report a higher discrimination capacity [3,10,21]. This suggests that in our population other not considered factors may play an important role in predicting CD. In terms of predicting the probability of a CD, the fit of all models was imperfect as reported by other authors [10].

In our study malpresentation, cord prolapse, placenta praevia and placental abruption proved to be the most important risk factors for cesarean delivery. This finding is consistent with prior epidemiological studies and clinical knowledge [6] and supports the predictive validity of the models.

A significant difference in terms of discrimination and fit was observed between model 2 and 3. The latter includes clinical variables retrieved from BC: experience of previous abortion or stillbirth, fetal weight, fetal malformation, gestational age and parity. None of these variables are absolute indications for caesarean delivery but they are often included in risk adjustment models by other authors because they are important and frequent predictors of CD [9,11]. In our sample, 10% of the women did not deliver at term, 13% delivered a low, very low or high weight baby, 16% had a previous experience of previous abortion or stillbirth and 40% were multipara. Only fetal malformations occurred rarely (less than 1%). In particular, two of these variables, gestational age and parity are considered discriminatory when classifying deliveries according to the 10 Robson classification groups [22].

We found that adding variables retrieved from previous hospitalizations did not modify the performance of the models substantially. Cesarean section does not seem to be predicted by comorbidities registered in previous hospitalizations. These co-morbidities may have resolved and do not represent predictive factors any more or are sufficiently explained by comorbidities reported in the index hospitalization. Women who deliver are generally healthy and comorbidities present during delivery are probably sufficient to describe health problems.

Maternal and paternal education, citizenship and marital status proved to contribute very little to the model fit. The only study that examined the impact of socio-demographic variables [5] found that race and ethnicity do not affect risk adjustment models for cesarean delivery.

Our study also shows that even if the models with more variables explain more variation in the proportion of cesarean deliveries, at hospital level simple adjustment work as well as the complex one: hospital O/E ratios based on the most parsimonious model (model 1) correlated very well with the O/E ratios obtained using the most comprehensive set of variables (model 4).

Similarly Dimick et al. [20] studied risk adjustment models for surgical procedures and found that predictive models with a different number of variables had a very similar discrimination, good calibration and that hospital specific indicators resulting from parsimonious models were highly correlated [20].

This is a large population study, with very recent data. It relies on administrative data, which despite issues regarding their validity, are the most feasible option for searching variation in clinical practices, health outcomes and quality of care [14,16]. Previous work [14] suggests that administrative data may be equally discriminatory compared with data abstracted from clinical charts with respect to key outcomes. A limitation of this study is the potential lack of generalizability to other settings: the variables needed in adjustment models may not be the same if the quality of the coding is different or if databases include different sets of variables than those included in our HDR and BC. Our additional analyses excluding preterm gestation and other conditions such as herpes, antepartum hemorrhage, placental abruption, placenta previa or malpresentations showed, for instance, that homogenized populations yield a poorer predictive ability of the model (because the most important risk factors and indications for CD are omitted) but very similar O/E ratios across models.

The identification of the best predictive models is only the first step when applying risk adjustment procedures; variables in predictive models might be homogeneously distributed across hospitals and therefore are not actual confounders even if strongly associated with the outcome.

## Conclusions

This study highlights the need to consider the potential tradeoff between the accuracy of risk adjustment, the efficiency of data collection and the precision of the estimate. Undoubtedly, adding information from BC (parity, gestational age, previous stillbirth, fetal characteristics) to that available from HDR provides the most accurate data for risk adjustment. However, linkage between different datasets might be unfeasible or lead to a high proportion of missing data and potential selection bias. We argue that when the focus is on hospitals, using only demographic and clinical information from HDR for risk adjustment is a very reasonable option, given the high correlation of O/E ratios obtained between the most parsimonious models and the full model.

## Competing interests

The authors declare that they have no competing interests.

## Authors’ contributions

ES participated in the design of the study, performed the statistical analysis and drafted the manuscript. PR performed the statistical analysis and revised the manuscript critically. EC helped to perform the statistical analysis, contributed to the interpretation of data and revised the manuscript critically. GP contributed to the acquisition of data, and drafted the manuscript. MPF conceived the study, participated in its design and coordination, contributed to the interpretation of data and revised the manuscript critically. All authors read and approved the final manuscript.

## Pre-publication history

The pre-publication history for this paper can be accessed here:

http://www.biomedcentral.com/1472-6963/13/13/prepub

## Supplementary Material

Additional file 1 Variables of the four models and source of information. Click here for file

Additional file 2 Frequency of demographic and clinical characteristics and adjusted odds ratios (aOR) and 95% confidence intervals (CI) of cesarean delivery obtained with the four models. Click here for file
