# Supplementary material for: Risk adjustment for cesarean delivery rates: how many variables do we need? An observational study using administrative databases
Source: BMC Health Serv Res. 2013 Jan 10;13:13. doi: 10.1186/1472-6963-13-13 (PMC3554564; doi:10.1186/1472-6963-13-13)
Supplement: Additional file 1 — Variables of the four models and source of information. [file 1472-6963-13-13-S1.pdf]

Additional file 1 - Variables of the four models and source of information

Type of variable	source	Model 1	Model 2	Model 3	Model 4
**Socio-demographic**	**HDR**	age	age	age	Age
**Socio-demographic**	**BC**				Citizenship<br>Marital status<br>Education of mother, father
**Clinical**	**HDR**	HIV<br>Diabetes<br>Hypertension<br>Lung<br>Thyroid diseases<br>Genital Herpes<br>Other severe comorbidities of the mother<br>Substance abuse<br>Ante-partum haemorrhage / abruption / Placenta Praevia<br>Eclampsia/Pre-eclamps<br>Cephalopelvic disproportion<br>RH-Isoimmunisation<br>Polyhydramnios<br>Oligohydramnios<br>Premature rupture of the membranes<br>Other problems of the amnios<br>Cord prolapse<br>Abortion threads /assisted fecundation<br>Intrauterine growth retardation<br>Multiple pregnancy<br>Malpresentation<br>Foetal abn affecting mother	HIV<br>Diabetes<br>Hypertension<br>Lung<br>Thyroid diseases<br>Genital Herpes<br>Other severe comorbidities of the mother<br>Substance abuse<br>Ante-partum haemorrhage / abruption / Placenta Praevia<br>Eclampsia/Pre-eclamps<br>Cephalopelvic disproportion<br>RH-Isoimmunisation<br>Polyhydramnios<br>Oligohydramnios<br>Premature rupture of the membranes<br>Other problems of the amnios<br>Cord prolapse<br>Abortion threads /assisted fecundation<br>Intrauterine growth retardation<br>Multiple pregnancy<br>Malpresentation<br>Foetal abn affecting mother	HIV<br>Diabetes<br>Hypertension<br>Lung<br>Thyroid diseases<br>Genital Herpes<br>Other severe comorbidities of the mother<br>Substance abuse<br>Ante-partum haemorrhage / abruption / Placenta Praevia<br>Eclampsia/Pre-eclamps<br>Cephalopelvic disproportion<br>RH-Isoimmunisation<br>Polyhydramnios<br>Oligohydramnios<br>Premature rupture of the membranes<br>Other problems of the amnios<br>Cord prolapse<br>Abortion threads /assisted fecundation<br>Intrauterine growth retardation<br>Multiple pregnancy<br>Malpresentation<br>Foetal abn affecting mother	HIV<br>Diabetes<br>Hypertension<br>Lung<br>Thyroid diseases<br>Genital Herpes<br>Other severe comorbidities of the mother<br>Substance abuse<br>Ante-partum haemorrhage / abruption / Placenta Praevia<br>Eclampsia/Pre-eclamps<br>Cephalopelvic disproportion<br>RH-Isoimmunisation<br>Polyhydramnios<br>Oligohydramnios<br>Premature rupture of the membranes<br>Other problems of the amnios<br>Cord prolapse<br>Abortion threads /assisted fecundation<br>Intrauterine growth retardation<br>Multiple pregnancy<br>Malpresentation<br>Foetal abn affecting mother
	**BC**			Previous abortion, previous still dead<br>Foetal weight<br>Foetal malformation<br>Gestational age<br>Parity	Previous abortion, previous still dead<br>Foetal weight<br>Foetal malformation<br>Gestational age<br>Parity
**Clinical**	**HDR previous hospitalization**		HIV<br>Diabetes<br>Hypertension<br>Lung<br>Thyroid diseases<br><br>Other severe comorbidities of the mother	HIV<br>Diabetes<br>Hypertension<br>Lung<br>Thyroid diseases<br><br>Other severe comorbidities of the mother	HIV<br>Diabetes<br>Hypertension<br>Lung<br>Thyroid diseases<br><br>Other severe comorbidities of the mother
