# Supplementary material for: Risk adjustment for cesarean delivery rates: how many variables do we need? An observational study using administrative databases
Source: BMC Health Serv Res. 2013 Jan 10;13:13. doi: 10.1186/1472-6963-13-13 (PMC3554564; doi:10.1186/1472-6963-13-13)
Supplement: Additional file 2 — Frequency of demographic and clinical characteristics and adjusted odds ratios (aOR) and 95% confidence intervals (CI) of cesarean delivery obtained with the four models. [file 1472-6963-13-13-S2.pdf]

Additional file 2 - Frequency of demographic and clinical characteristics and adjusted odds ratios (aOR) and 95% confidence intervals (CI) of cesarean delivery obtained with the four models

			Model 1		Model 2		Model 3		Model 4	
Variables	N	%	aOR	CI	aOR	CI	aOR	CI	aOR	CI
Maternal age										
<18	315	0.36	0.64	(0.44 - 0.92)	0.63	(0.44 - 0.92)	0.40	(0.27 - 0.58)	0.34	(0.23 - 0.49)
18-24	10,544	12.04	0.77	(0.72 - 0.83)	0.77	(0.72 - 0.83)	0.59	(0.55 - 0.63)	0.54	(0.50 - 0.58)
25-29	20,234	23.11	0.88	(0.84 - 0.93)	0.88	(0.84 - 0.93)	0.79	(0.74 - 0.83)	0.76	(0.72 - 0.81)
30-34	31,111	35.53	Ref		Ref		Ref		Ref	
35-39	20,702	23.64	1.18	(1.13 - 1.24)	1.18	(1.13 - 1.24)	1.40	(1.33 - 1.48)	1.41	(1.34 - 1.48)
<39	4,668	5.33	1.82	(1.68 - 1.97)	1.82	(1.68 - 1.97)	2.38	(2.18 - 2.59)	2.36	(2.17 - 2.57)
Citizenship										
Italian	64,661	73.84							Ref	
High income excluding Italy*	884	1.01							0.73	(0.59 - 0.91)
Low income	22,029	25.15							1.03	(0.98 - 1.09)
Maternal education										
University	20,757	23.70							0.82	(0.77 - 0.87)
High school	39,996	45.67							Ref	
Middle school	23,606	26.96							0.86	(0.82 - 0.90)
Primary	3,215	3.67							1.09	(0.97 - 1.22)
Marital status										
Married	58,002	66.23							Ref	
Not declared	3,928	4.49							0.89	(0.80 - 0.98)
Single	23,476	26.81							1.05	(1.01 - 1.10)
Separated/divorced	2,034	2.32							1.13	(0.99 - 1.29)
Widow	134	0.15							1.33	(0.81 - 2.17)
HIV	76	0.09	66.50	(26.50 - 166.88)	65.95	(28.48 - 152.72)	83.84	(35.19 - 199.77)	78.13	(32.78 - 186.26)
Diabetes	1,184	1.35	2.17	(1.89-2.49)	2.17	(1.89 - 2.49)	2.29	(1.98 - 2.64)	2.22	(1.93 - 2.57)
Hypertension	1,787	2.04	3.13	(2.81 - 3.48)	3.15	(2.83 - 3.50)	2.67	(2.39 - 2.99)	2.65	(2.36 - 2.96)
Lung diseases	100	0.11	10.02	(5.50 - 18.24)	5.52	(3.46 - 8.78)	5.47	(3.30 - 9.06)	5.24	(3.16 - 8.71)
Other severe comorbidities of the mother	839	0.96	4.92	(4.05 - 5.97)	3.23	(2.77 - 3.76)	3.05	(2.59 - 3.60)	3.06	(2.60 - 3.61)
Substance abuse	24	0.03	3.95	(1.66 - 9.41)	3.96	(1.67- 9.44)	3.45	(1.44 - 8.25)	3.26	(1.36 - 7.79)
Eclampsia or pre-eclampsia	1,282	1.46	10.64	(9.34 - 12.11)	10.66	(9.37 - 12.13)	7.33	(6.38 - 8.43)	7.32	(6.37 - 8.42)

Antepartum hemorrhage/placental abruption/placenta previa	1,111	1.27	102.40	(77.54 - 135.24)	102.30	(77.46 - 135.12)	109.78	(82.46 - 146.13)	110.43	(82.95 - 147.00)
Cephalopelvic disproportion	1,045	1.19	13.43	(11.70 - 15.42)	13.47	(11.73 - 15.47)	13.60	(11.67 - 15.83)	13.57	(11.65 - 15.81)
Isoimmunization	727	0.83	0.69	(0.55 - 0.88)	0.70	(0.55 - 0.88)	0.73	(0.57 - 0.93)	0.74	(0.58 - 0.94)
Polyhydramnios	201	0.23	3.68	(2.65 - 5.11)	3.68	(2.65 - 5.11)	4.33	(3.03 - 6.17)	4.22	(2.95 - 6.02)
Oligohydramnyos	3,330	3.80	1.85	(1.70 - 2.01)	1.84	(1.69 - 2.01)	1.64	(1.50 - 1.79)	1.63	(1.49 - 1.78)
Other problems of the amnios	83	0.09	8.29	(5.06 - 13.59)	8.14	(5.00 - 13.44)	6.36	(3.78 - 10.69)	6.40	(3.81 - 10.76)
Cord prolapse	60	0.07	329.48	(45.43-2389.65)	330.42	(45.56 - 2396.37)	623.81	(84.39 - 4611.20)	623.78	(84.40 - 4610.26)
Premature rupture of membrane	13,228	15.10	0.81	(0.76 - 0.85)	0.81	(0.76 - 0.85)	0.66	(0.62 - 0.70)	0.65	(0.62 - 0.69)
Abortion threaten	289	0.33	5.01	(3.71 - 6.76)	5.01	(3.71 - 6.75)	2.78	(2.00 - 3.87)	2.77	(1.99 - 3.85)
Multiple birth	1,315	1.50	23.32	(19.51 - 27.87)	23.29	(19.48 - 27.83)	16.50	(13.55 - 20.09)	16.72	(13.73 - 20.37)
Intrauterine growth retard	1,349	1.54	6.14	(5.41 - 6.97)	6.14	(5.40 - 6.96)	3.23	(2.79 - 3.74)	3.24	(2.79 - 3.75)
Fetal abn affecting mother	295	0.34	5.30	(4.14 - 6.80)	5.32	(4.15 - 6.81)	3.89	(2.93 - 5.16)	3.94	(2.96 - 5.23)
Malpresentation	4,317	4.93	227.86	(186.20 - 278.85)	228.08	(186.37 - 279.12)	259.22	(211.19 - 318.17)	259.98	(211.81 - 319.11)
Gestational age		0.00								
Term	78,818	90.00					Ref		Ref	
Preterm	6,432	7.34					1.61	(1.48 - 1.76)	1.60	(1.47 - 1.75)
Post-term	2,324	2.65					1.31	(1.17 - 1.47)	1.31	(1.17 - 1.47)
Neonatal weight		0.00								
Normal	75,972	86.75					Ref		Ref	
Very low	766	0.87					4.86	(3.78 - 6.24)	4.81	(3.75 - 6.19)
Low	4,282	4.89					1.75	(1.58 - 1.93)	1.74	(1.58 - 1.92)
Overweight	6,554	7.48					1.19	(1.10 - 1.28)	1.19	(1.10 - 1.28)
Congenital malformation	818	0.93					1.60	(1.32 - 1.94)	1.60	(1.32 - 1.94)
Multiparity	34,721	39.65					0.21	(0.20 - 0.22)	0.20	(0.19 - 0.21)
Previous stillbirth	14,288	16.32					1.12	(1.06 - 1.18)	1.12	(1.06 - 1.18)

*High-income countries have been defined as in the World Bank Country classification (http://data.worldbank.org/about/country-classifications)
